# Supplementary material for: Evaluating the Impact of Needle-Free Delivery of Inactivated Polio Vaccine on Nigeria’s Routine Immunization Program: An Implementation Hybrid Trial
Source: Vaccines (Basel). 2025 May 16;13(5):533. doi: 10.3390/vaccines13050533 (PMC12116027; doi:10.3390/vaccines13050533)
Supplement: Supplementary file 1 [file vaccines-13-00533-s001.zip › vaccines-3592559-supplementary.pdf]

Supplemental Materials: Appendix A

Table S1. Baseline characteristics of children from household survey.

	**Control**		**Intervention**		**Total**
	n	%	N	%	N
Total	1842	53.7	1591	46.3	3433
State					
Kano	1018	53.5	885	46.5	1903
Oyo	824	53.9	706	46.1	1530
LGA					
Atiba	120	43.3	157	56.7	277
Gwale	194	60.6	126	39.4	320
Ibadan North	253	87.5	36	12.5	289
Iseyin	213	39	333	61	546
Kabo	609	73.9	215	26.1	824
Kura	215	28.3	544	71.7	759
Ogo Oluwa	238	56.9	180	43.1	418
Wealth Quintiles					
Poorest	502	63.8	285	36.2	787
Poorer	292	50.1	291	49.9	583
Middle	370	50.5	362	49.5	732
Richer	340	48.8	357	51.2	697
Richest	338	53.3	296	46.7	634
Education					
Primary or less	337	58.4	240	41.6	577
Secondary & Higher	1073	51.7	1004	48.3	2077
Quranic	432	55.5	347	44.5	779
Polygamous household					
No	1409	54.2	1193	45.8	2602
Yes	433	52.1	398	47.9	831
Gender of head of Household					
Male	1803	53.4	1571	46.6	3374
Female	39	66.1	20	33.9	59

Place of delivery					
Home / on the way	916	54.3	771	45.7	1687
Facility	926	53	820	47	1746
Residence					
Rural	1190	52.8	1065	47.2	2255
Urban	652	55.3	526	44.7	1178
Distance from apex facility					
<2 KM	1146	62.2	1131	71.1	2277
2-5 KM	563	30.6	178	11.2	741
5-10 KM	92	5	110	6.9	202
>10 KM	41	2.2	172	10.8	213
Facility size (average monthly volume of vaccinations)					
<20/month	156	38.2	252	61.8	408
20-50/month	1253	55.8	992	44.2	2245
>50/month	433	55.5	347	44.5	780
Early childhood vaccinations received					
BCG	n	Col%	N	Col%	N
No	213	11.6	214	13.5	427
Yes	1629	88.4	1377	86.5	3006
Hep0					
No	505	27.4	473	29.7	978
Yes	1337	72.6	1118	70.3	2455
OPV0					
No	301	16.3	303	19	604
Yes	1541	83.7	1288	81	2829
IPV1					
No	290	15.7	281	17.7	571
Yes	1552	84.3	1310	82.3	2862

Table S2. Percentage of respondents reporting receipt of Inactivated Polio Vaccine using Tropis

	Overall			Control			Intervention		
	N	%	N	n	%	N	N	%	N
Total	710	26	2728	83	5.6	1475	627	50	1253
State									

Kano	293	19.8	1483	25	3.1	807	268	39.6	676
Oyo	417	33.5	1245	58	8.7	668	359	62.2	577
LGA									
Atiba	62	29.2	212	5	5.4	93	57	47.9	119
Gwale	46	18.2	253	8	5.4	147	38	35.8	106
Ibadan North	19	8.1	235	5	2.4	207	14	50	28
Iseyin	222	47.6	466	39	20.6	189	183	66.1	277
Kabo	66	10.4	635	4	0.8	480	62	40	155
Kura	181	30.4	595	13	7.2	180	168	40.5	415
Ogo Oluwa	114	34.3	332	9	5	179	105	68.6	153
Wealth Quintiles									
Poorest	75	13	577	7	1.8	387	68	35.8	190
Poorer	97	22.1	438	0	0	214	97	43.3	224
Middle	162	28.8	562	16	5.9	271	146	50.2	291
Richer	186	32	581	26	8.9	292	160	55.4	289
Richest	190	33.3	570	34	10.9	311	156	60.2	259
Education									
Primary or less	88	21.6	408	5	2.1	239	83	49.1	169
Secondary & Higher	531	30.4	1746	72	7.9	909	459	54.8	837
Quranic	91	15.9	574	6	1.8	327	85	34.4	247
Polygamous household									
No	576	27.5	2095	68	5.9	1144	508	53.4	951
Yes	134	21.2	633	15	4.5	331	119	39.4	302
Gender of head of Household									
Male	701	26.2	2680	82	5.7	1443	619	50	1237
Female	9	18.8	48	1	3.1	32	8	50	16

Residence									
Rural	449	25.6	1754	59	6.2	944	390	48.1	810
Urban	261	26.8	974	24	4.5	531	237	53.5	443
Facility size (Average monthly volume of vaccinations)									
20-50/month	423	23.4	1806	32	3.2	1009	391	49.1	797
<20/month	116	37.7	308	8	7.5	107	108	53.7	201
>50/month	171	27.9	614	43	12	359	128	50.2	255

Table S3. *Coverage of other antigens among caregivers present during vaccination across study arms (Per Protocol)*

	**Control**		**Intervention**		**Total**
	N	%	N	%	
Rota1					
No	71	5.1	36	5.7	107
Yes	1321	94.9	591	94.3	1912
Rota2					
No	221	15.9	108	17.2	329
Yes	1171	84.1	519	82.8	1690
Rota3					
No	482	34.6	233	37.2	715
Yes	910	65.4	394	62.8	1304
Measles1					
No	1173	84.3	545	86.9	1718
Yes	219	15.7	82	13.1	301
Penta1					
No	31	2.2	1	0.2	32
Yes	1361	97.8	626	99.8	1987
Penta3					
No	379	27.2	118	18.8	497
Yes	1013	72.8	509	81.2	1522
Pneu3					
No	379	27.2	121	19.3	500
Yes	1013	72.8	506	80.7	1519
